# Case Report: A Novel Compound Heterozygote Mutation of the *SCNN1B* Gene Identified in a Chinese Familial Pseudohypoaldosteronism Disease Type I With Persistent Hyperkalemia

**DOI:** 10.3389/fped.2022.831284

**Published:** 2022-03-10

**Authors:** Zongzhi Liu, Xiaojiao Wang, Zilong Zhang, Zixin Yang, Junyun Wang, Yajuan Wang

**Affiliations:** ^1^Shenzhen Key Laboratory of Synthetic Genomics, Guangdong Provincial Key Laboratory of Synthetic Genomics, CAS Key Laboratory of Quantitative Engineering Biology, Shenzhen Institute of Synthetic Biology, Shenzhen Institutes of Advanced Technology, Chinese Academy of Sciences, Shenzhen, China; ^2^Central Laboratory, National Cancer Center/National Clinical Research Center for Cancer/Cancer Hospital and Shenzhen Hospital, Chinese Academic of Medical Sciences and Peking Union Medical College, Shenzhen, China; ^3^Department of Neonatal Center, Beijing Children's Hospital, Capital Medical University, National Center for Children's Health, Beijing, China; ^4^Tianjin Novogene Bioinformatic Technology Co., Ltd., Tianjin, China; ^5^CAS Key Laboratory of Genome Sciences and Information, China National Center for Bioinformation, Beijing Institute of Genomics, Chinese Academy of Sciences, Beijing, China; ^6^Department of Neonatology, Children's Hospital, Capital Institute of Pediatrics, Beijing, China

**Keywords:** pseudohypoaldosteronism type I, whole-exome sequencing, *SCNN1B*, frameshift mutations, genetic pattern, rare disease

## Abstract

**Background:**

Pseudohypoaldosteronism (PHA) diseases are difficult to diagnose because symptoms are often non-specific and an in-depth pathogenesis study is still lacking.

**Case Presentation:**

We present the case of a 19-day-old neonate who presented with unexplained recurrent hyperkalaemia, hypovolemia and metabolic acidosis, whose parents did not have significant clinical disease characteristics. Whole-exome sequencing was performed to confirm the disease and genetic pattern of the neonate. Sanger sequencing was performed to identify the mutation sites. Secondary structure comparisons and 3D model construction were used to predict changes in protein structure. Two novel frameshift mutations in the *SCNN1B* gene were identified (c.1290delA and c.1348_1361del), which resulted in amino acid synthesis termination (p.Gln431ArgfsTer2 and p.Thr451AspfsTer6). Considering the clinical phenotype and genetic analysis, this case was finally identified as a PHA type I disease. Genetic analysis showed that the neonate suffered complex heterozygosity in the *SCNN1B* gene inherited from the parents, which is passed on in an autosomal recessive inheritance pattern. These two deleterious mutations resulted in an incomplete protein 3D structure.

**Conclusions:**

Our results have confirmed the associations of mutations in the SCNN1B gene with recurrent hyperkalaemia, which can cause severe PHA type I disease, meanwhile suggested clinical attention should be paid when persistent recurrent hyperkalemia is accompanied by these types of mutations.

## Introduction

Pseudohypoaldosteronism (PHA) is a severe disease first described by Cheek and Perry (1), characterized by congenital resistance to the action of aldosterone on epithelial tissue, failure to thrive, hyperkalaemia, hypovolemia, and metabolic acidosis. PHAs can be divided into types I and II. PHA type I often harbors mutations in the *SCNN1A, SCNN1B*, and *SCNN1G* genes, which encode the epithelial sodium channel (ENaC) (2, 3). PHA type II is caused by mutations in the *WNK1* and *WNK4* genes, which encode lysine-deficient protein kinases. PHA type I results in excessive salt wasting despite very high plasma aldosterone and renin levels, whereas PHA type II leads to blood pressure disorder-related diseases (4–6). Molecular level research on PHA type I shows the *SCNN1A* (12p13), *SCNN1B* (16p12.2–p12.1), and *SCNN1G* (16p12) genes encoding the three homologous α, β, and γ subunits of ENaCs (7–10), which are membrane-bound ion channels that are selectively permeable to Na^+^ ions. Changes in Na? concentration affect the movement of fluids and, consequently, fluid volume and blood pressure (11, 12). ENaC activity is modulated by the mineralocorticoid aldosterone, and the α, β, and γ subunits of ENaC are essential for transport to the membrane assembly of functional channels on the membrane; mutations in *SCNN1A, SBNN1B*, and *SCNN1G* lead to loss of ENaC activity (13–15).

Here, we report the case of a 19-day-old neonate presumptively diagnosed with PHA with two novel frameshift mutations in *SCNN1B* (c.1290delA and c.1348_1361del), which resulted in premature amino acid synthesis termination (p.Gln431ArgfsTer2 and p.Thr451AspfsTer6) by both two chromosomes. We also reviewed and analyzed the clinical features of the neonate to further clarify the correlation between the deletion region and the phenotype.

## Materials and Methods

### Editorial Policies and Ethical Considerations

This study was agreed by the Research Ethical Review Committee, Beijing Children's Hospital Affiliated to Capital Medical University (approved protocol no.2017-k-81). Written informed consent was obtained from the individual(s), and minor(s)' legal guardian/next of kin, for the publication of any potentially identifiable images or data included in this article. All blood samples were obtained after written informed patient consent and were fully anonymized. The participants and legal guardian provided written informed consent to participate in this study.

### Clinical Presentation

A 19-day-old neonate suffered an inexplicable electrolyte disturbance for 15 days and was admitted to the neonatal intensive care unit at the Beijing Children's Hospital with a history of poor feeding, hypothermia, lethargy, and bradycardia. She was born by cesarean section after a full-term pregnancy with a birth weight of 3,490 g (10–90^th^ percentile) and lost 15% of her weight 5 days after birth. She was the first child of her parents, and the mother had no perinatal problems. Neither of the parents had a family history of significant illness or unexplained death.

On physical examination, her weight and height were 3.09 kg (<3^rd^ percentile) and 50.2 cm (3^rd^-10^th^ percentile), respectively, and her head circumference was 34.1 cm (10–50^th^ percentile). Routine examination showed a blood pressure of 73/39 mmHg, pulse rate of 148/min, respiratory rate of 44/min, and temperature of 37.2°C. The neonate was pale and lethargic and exhibited hypotonic extremities and severe dehydration. Her external genitals developed normally, and other physical examination results were within the normal range.

**Figure 1:**
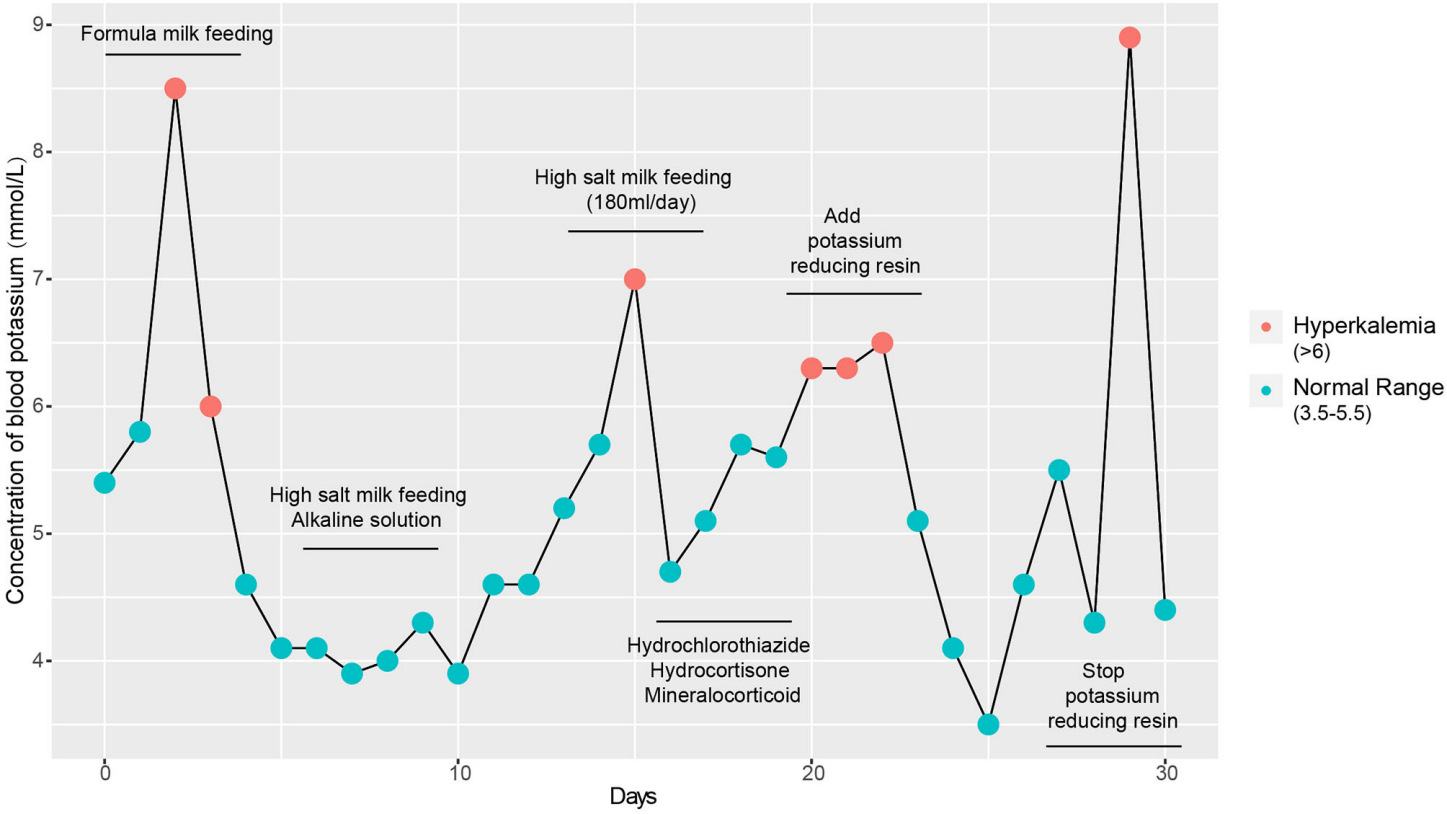
Detection of blood potassium from admission to discharge. Repeated clinical symptoms of hyperkalaemia in the infant from day 1 to day 30.

The initial serum laboratory examinations results were as follows: hyponatraemia: sodium 111 mmol/L (normal range = 130–150 mmol/L), hyperkalaemia: potassium 9.0 mmol/L (normal range = 3.5–5.5 mmol/L), hypochloraemia: chloride 83.6 mmol/L (normal range = 98–108 mmol/L), urea nitrogen 4.2 mmol/L (normal range = 1.7–7.1 mmol/L), and creatinine 23 μmol/L (normal range = 30–104 μmol/L). Arterial blood gas analysis revealed metabolic acidosis: pH = 7.227 (normal range = 7.251–7.429) and base excess:−14 mmol/L (normal range = −6–2). Plasma adrenocorticotropic hormone < 5–11.9 pg/ml(normal range = 0–46 pg/ml), Plasma cortisol 1.27–9.28 umol/dl (normal range =5–25 μmol/dl), Plasma testosterone < 20 ng/dl (normal range = 0–106.9), Plasma 17-α-hydroxyprogesterone 5.18 nmol/L (normal range = 0.31–38.1). Further laboratory studies subsequently revealed markedly increased plasma renin 5.36 ng/mL/h (normal range = 0.00–0.79 ng/mL/h), serum aldosterone 22.78 ng/dL (normal range = 5.9–17.4 ng/dL), and angiotensin II 398.7 pg/mL (normal range = 40.6–90.0 pg/mL). B-ultrasound of adrenal gland showed that the structure of adrenal gland was normal. Symptoms of hyperkalaemia persisted during the treatment from day 1 to day 4 (Figure 1). Meanwhile, through differential diagnosis, we ruled out renal failure, hypovolemia: shock or dehydration, adrenocortical insufficiency: adrenal hemorrhage, adrenal hypoplasia, etc., congenital adrenal hyperplasia, potassium retention diuretics and other diseases. Based on these clinical tests, a presumptive diagnosis of PHA was made (Table 1), metabolic acidosis was corrected, and blood potassium was lowered using simultaneous high-salt milk feeding (1 g Nacl/day, 20–30 ml/feeding). Because the PHA disease was not confirmed, the type and genetic pattern was also unclear, accurate treatment could not be provided; therefore, whole-exome sequencing and Sanger sequencing were performed on the neonate and her parents who without typical PHA disease symptoms.

**Table 1:** Differential diagnosis of the neonate.

**Pathogeny**	**Diagnosis**	**The neonate**
Excessive potassium intake	Intravenous infusion a large amount of potassium liquid	No
Renal potassium depletion disorder	Renal failure	No
	Hypovolemia: shock or dehydration	No
	Adrenocortical insufficiency: adrenal hemorrhage, adrenal hypoplasia, etc.	No
	Congenital adrenal hyperplasia	No
	Potassium retention diuretics	No
	Pseudohypoaldosteronism	Highly suspicious

### Genomic DNA Extraction

We obtained the peripheral blood of the neonate and her parents at Beijing Children's Hospital and extracted the DNA immediately using the QIAamp DNA Blood Mini Kit according to the manufacturer's instructions.

### Whole-Exome Sequencing and Single Nucleotide Polymorphism Calling

Exome capture was performed using the Agilent SureSelect Human All Exon V6, according to the manufacturer's standard protocols. The Illumina Hiseq 2500 platform was used to detect the DNA sequence, and paired-end sequencing was conducted with a read length of 150 bp (16). More than 10 GB of clean sequence data and more than 100 × read depth were obtained for each DNA sample. All individual sequence reads were aligned to the human reference genome (NCBI build GRH37/hg19) using the Burrows-Wheeler Aligner software (17). The genome analysis toolkit, SAMtools, and Picard tools were used for bioinformatic analysis, and SNPs and insertion-deletions (indels) were identified using the genome analysis toolkit (18, 19).

### 3D Modeling

Amino acid sequence alignment and the 3D structure of SCNN1B were constructed using Swiss-Model software (https://swissmodel.expasy.org/).

## Results

### Mutation Analysis and Verification

**Figure 2:**
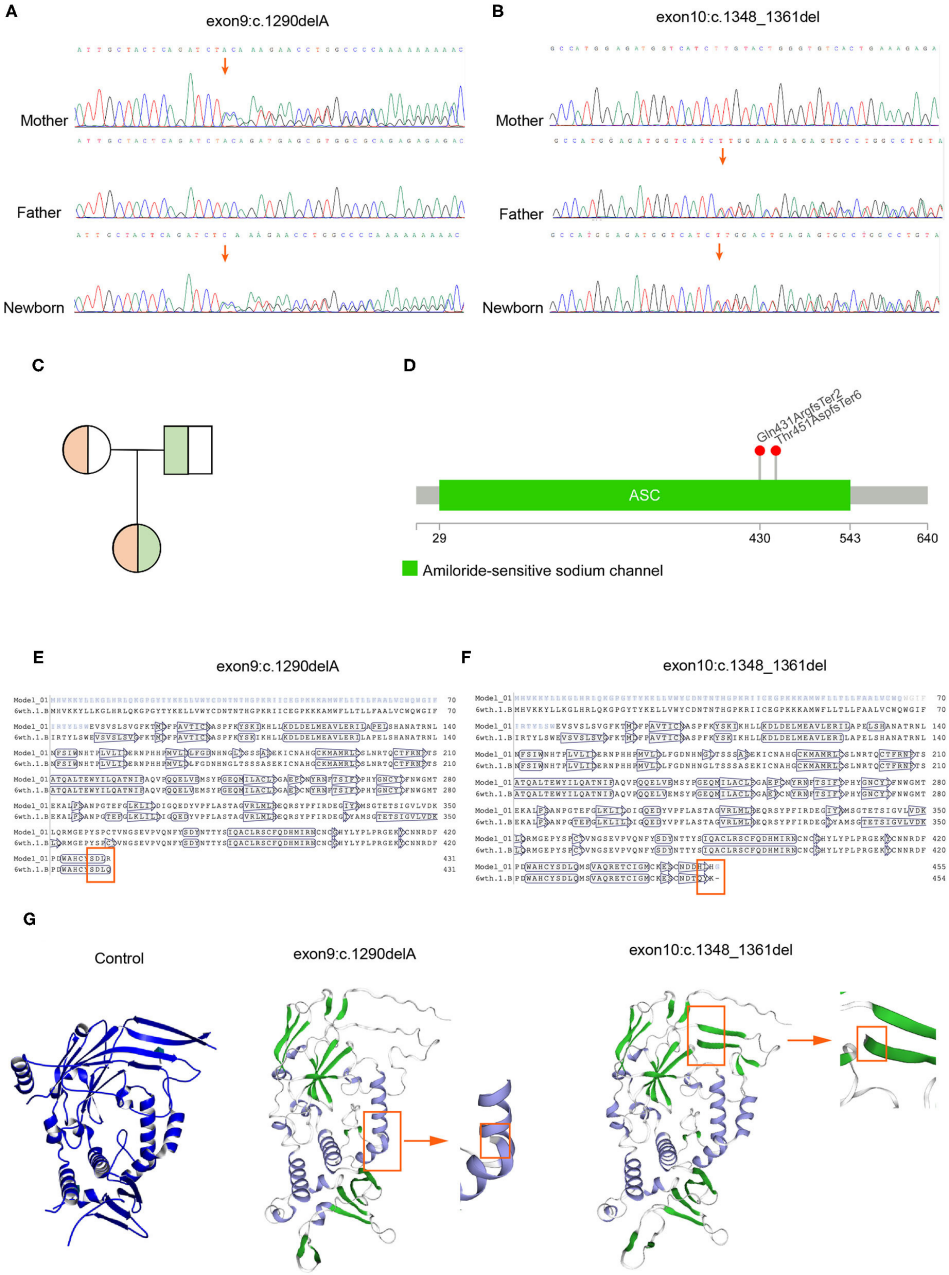
Mutation verification and protein 3D structure prediction. **(A)** Sanger sequencing demonstrated that c.1290delA was inherited from the mother. **(B)** Sanger sequencing demonstrated that c.1348_1361del was inherited from the father. **(C)** Genetic pattern analysis of compound heterozygotes. **(D)** Location of the mutation sites in the *SCNN1B* gene. **(E,F)** Amino acid sequence alignment indicated that both mutations caused early termination of amino acid synthesis (6wth.1.b refers to the amino acid sequence after mutation; Model_01 refers to the non-mutated amino acid sequence). **(G)** The 3D model predicted an incomplete ENaC structure after mutation.

In order to identify the PHA disease and the type of PHA, SNPs and indels in the *SCNN1A, SCNN1B, SCNN1G, WNK1*, and *WNK4* genes were investigated. As shown in Table 2, two novel frameshift mutations in *SCNN1B* (c.1290delA and c.1348_1361del) were revealed, both of which led to the production of the stop codon TGA. We further verified the two new mutations using Sanger sequencing (Figure 2A). Neither mutation had been described previously (http://www.ncbi.nlm.nih.gov/projects/SNP). Other candidate genes were also analyzed, but no significant pathogenic SNPs or indels were found (Table 2). Based on the molecular-level results, PHA type I was preliminarily identified.

**Table 2:** SNPs and indels analysis of SCNN1B and WNK1 in the neonate.

**Gene name**	**Chromosome position**	**RS number**	**Ref Allele**	**SNP allele**	**Functional consequence**	**Allele frequency**	**Global MAF**	**Clinical significance**
SCNN1B	16:23388504	-	TA	T	-	0.5	-	-
SCNN1B	16:23388650	-	TGACACCCAGTACAA	T	-	0.5	-	-
SCNN1B	16:23360199	rs238547	T	C	Synonymous codon	1	T:0.2115	Benign
WNK1	12:1017197	rs4766334	C	T	Synonymous codon	1	C:0.0130	Benign
WNK1	12:862989	rs3168640	T	C	Synonymous codon	1	T:0.0244	Benign
WNK1	12:987482	rs1012729	G	A	Synonymous codon	1	G:0.3205	Benign
WNK1	12:990912	rs956868	A	C	Missense	1	A:0.1472	Benign
WNK1	12:993930	rs7300444	C	T	Synonymous codon	1	T:0.3990	Benign
WNK1	12:994487	rs7955371	G	C	Synonymous codon	1	G:5008:0.0132	Benign
WNK1	12:862641	rs3088353	T	G	5'utr variant	0.5	G:5008:0.3399	Benign
WNK1	12:939302	rs10774466	A	G	Synonymous codon	1	A:5008:0.3065	Benign

### Confirmation of Compound Heterozygosity and Genetic Pattern

Because the neonate's parents do not have typical PHA disease characteristics, next, we explored the genetic patterns of these mutations. As shown in Figures 2A–C, c.1290delA was carried by her mother, whereas c.1348_1461del was carried by her father, and the neonate inherited these two frameshift mutations concurrently. Her parents possessed heterozygous mutations with a normal phenotype, indicating a recessive inheritance pattern (Figure 2C). Thereafter, we confirmed that these two mutations were distributed in different chromosomes in the neonate, so the translation of the *SCNN1B* gene was terminated prematurely in both chromosomes. Whole-exome sequencing results showed that c.1290delA occurred at chr16:23388505, whereas c.1348_1461del occurred at chr16:23388651, and the distance between these two mutations was 146 bp (Figure 2D). We then counted the number of reads covered in this range, there was no single read containing both mutations, all the reads covered from chr16:23388505 to chr16:23388651 had only one mutation, either c.1290delA or c.1348_1461del, which indicates that the two mutations occurred in different chromosomes. Based on these findings, the neonate suffered a “double-stop” compound heterozygous mutation in the *SCNN1B* gene, which led to no functional B-subunit protein of ENaC and severe and persistent electrolyte abnormalities.

### Amino Acid Sequence Alignment and 3D Prediction of Protein Structure

Further, we want to know whether these two mutations will cause changes in the three-dimensional structure of the protein. We found that the two mutations at the amino acid level were p.Gln431ArgfsTer2 and p.Thr451AspfsTer6. After amino acid sequence alignment, we found that these two frameshift mutations led to the early termination of amino acid synthesis (Figures 2E,F). We also found that both mutations led to an incomplete structure in the protein 3D prediction model (Figure 2G).

### Follow Up and Outcome

After confirmation of recessive hereditary PHA type I using genetic analysis, the parents were asked to go back to the local hospital for treatment after a clear diagnosis, we recommend continuing oral potassium lowering resin (1–2g/kg/day) and high-salt milk feeding (1 g Nacl/day, 20–30 ml/feeding) as therapeutic approach. Serum electrolytes returned to the normal range after initial parenteral hydration, oral sodium supplementation, and kayexalate usage; all clinical indicators were within the normal range after returning to the local hospital, but the development level was slightly slower than that of normal infants. At 3 months of age, the following measurements were obtained: weight 3.8 kg (normal range = 4.4–8.71 kg), height 55 cm (normal range = 54.2–67.5 cm), and head circumference 37 cm (normal range = 37.4–42.2 cm). At the age of 4 months, the infant returned to Beijing Children's Hospital due to convulsions without obvious causes, respiratory infection symptoms, increased blood potassium level, decreased blood sodium level, and ventricular tachycardia. Subsequently, resuscitative efforts were unsuccessful and the infant died.

## Discussion

PHA is a mineralocorticoid resistance disease characterized by renal salt wasting, dehydration, and failure to thrive. PHA type I typically presents in the neonatal period with severe salt-wasting crisis with hyponatraemia, hyperkalaemia, acidosis, and dehydration due to sodium loss through the kidneys, colon, sweat, and salivary glands (20, 21). Patients may also present with lethargy and failure to thrive. PHA type I is often recessively inherited, and genetic mutations cause manifold loss of function of ENaC protein subunits and sodium reabsorption disfunction (3, 22). There is no specific treatment for PHA type I, except for the potassium ion exchange resin.

We present a case of PHA type I identified using whole-exome sequencing, with two novel frameshift mutations in the *SCNN1B* gene, which resulted in severe hyperkalaemia, hyponatraemia, metabolic acidosis, moreover, the emergence of compound heterozygotes of this mutant type may lead to growth retardation and early neonatal mortality. These mutations were inherited from the parents, who did not exhibit typical PHA clinical symptoms. Although several cases of patients with PHA type I have previously been presented, and mutations such as c.1466 +1 G > A, c.1288delC, and c.1266-1G > C have been reported (9, 23–26). This neonate carried novel compound heterozygote mutations in the *SCNN1B* gene, both of which resulted in an abnormal stop codon.

The ENaC plays a critical role in cellular functions, such as Na^+^ and water balance, blood pressure stabilization, and response to aldosterone. As a result, mutations in ENaC genes generally produce clinical symptoms of failure to thrive, hypovolemia caused by sodium wasting, metabolic acidosis, and severe hyperkalaemia.c.1290delA and c.1348_1461del changed the reading frame, resulting in a completely different translation compared with the original (27, 28), and these two deletions lead to abnormal stop codons that were distributed in different chromosome. These two novel frameshift mutations led to p.Gln431ArgfsTer2 and p.Thr451AspfsTer6 (29). The advantage of our study is we established the mutations identified were inherited from both parents, who had novel frameshift mutations. The parents were not diagnosed with PHA because the mutation in the *SCNN1B* gene only occurred in one of the homologous chromosome. The other homologous chromosome had a *SCNN1B* gene with normal structure and function; therefore, the parents did not exhibit significant clinical symptoms. However, both of them carried different mutations in the *SCNN1B* gene, and their daughter inherited these mutations.

This case increased our understanding of phenotypes resulting from *SCNN1B* mutations. Our results highlight the need for increased attention to parents or children with such genotypes in clinical practice.

## Data Availability Statement

The original contributions presented in the study are included in the article/supplementary files, further inquiries can be directed to the corresponding authors.

## Ethics Statement

The studies involving human participants were reviewed and approved by Research Ethical Review Committee, Beijing Children's Hospital Affiliated to Capital Medical University (Approved Protocol No. 2017-k-81). Written informed consent to participate in this study was provided by the participants' legal guardian/next of kin. Written informed consent was obtained from the individual(s), and minor(s)' legal guardian/next of kin, for the publication of any potentially identifiable images or data included in this article.

## Author Contributions

ZL analyzed the data. XW and ZY collected the samples and treated the patient. ZZ contributed reagents, materials, and analysis tools. JW and YW participated in writing and correction. All authors read and approved the final manuscript.

## Funding

This work was supported by the National Natural Science Foundation of China (Grant Nos. 22005343 and 81702796), the Precision Medicine Research Program of the Chinese Academy of Sciences (Grant No. KJZD-EW-L14), the Sanming Project of Medicine in Shenzhen (Grant Nos. SZSM201812062 and SZSM201612097), and Shenzhen Key Medical Discipline Construction Fund (Grant No. SZXK075).

## Conflict of Interest

ZZ was employed by company Tianjin Novogene Bioinformatic Technology Co., Ltd., China. The remaining authors declare that the research was conducted in the absence of any commercial or financial relationships that could be construed as a potential conflict of interest.

## Publisher's Note

All claims expressed in this article are solely those of the authors and do not necessarily represent those of their affiliated organizations, or those of the publisher, the editors and the reviewers. Any product that may be evaluated in this article, or claim that may be made by its manufacturer, is not guaranteed or endorsed by the publisher.
